# Magnetic resonance imaging biomarkers for the early diagnosis of Alzheimer's disease: a machine learning approach

**DOI:** 10.3389/fnins.2015.00307

**Published:** 2015-09-01

**Authors:** Christian Salvatore, Antonio Cerasa, Petronilla Battista, Maria C. Gilardi, Aldo Quattrone, Isabella Castiglioni, the Alzheimer's Disease Neuroimaging Initiative

**Affiliations:** ^1^Institute of Molecular Bioimaging and Physiology, National Research Council (IBFM-CNR)Milan, Italy; ^2^Neuroimaging Research Unit, Institute of Molecular Bioimaging and Physiology, National Research Council (IBFM-CNR)Catanzaro, Italy; ^3^Department of Medical Sciences, Institute of Neurology, University “Magna Graecia”Catanzaro, Italy

**Keywords:** Alzheimer's disease, mild cognitive impairment, magnetic resonance imaging, support vector machine, structural neuroimaging biomarkers, machine learning, automatic classification, artificial intelligence

## Abstract

Determination of sensitive and specific markers of very early AD progression is intended to aid researchers and clinicians to develop new treatments and monitor their effectiveness, as well as to lessen the time and cost of clinical trials. Magnetic Resonance (MR)-related biomarkers have been recently identified by the use of machine learning methods for the *in vivo* differential diagnosis of AD. However, the vast majority of neuroimaging papers investigating this topic are focused on the difference between AD and patients with mild cognitive impairment (MCI), not considering the impact of MCI patients who will (MCIc) or not convert (MCInc) to AD. Morphological T1-weighted MRIs of 137 AD, 76 MCIc, 134 MCInc, and 162 healthy controls (CN) selected from the Alzheimer's disease neuroimaging initiative (ADNI) cohort, were used by an optimized machine learning algorithm. Voxels influencing the classification between these AD-related pre-clinical phases involved hippocampus, entorhinal cortex, basal ganglia, gyrus rectus, precuneus, and cerebellum, all critical regions known to be strongly involved in the pathophysiological mechanisms of AD. Classification accuracy was 76% AD vs. CN, 72% MCIc vs. CN, 66% MCIc vs. MCInc (nested 20-fold cross validation). Our data encourage the application of computer-based diagnosis in clinical practice of AD opening new prospective in the early management of AD patients.

## Introduction

The increase in life expectancy and the prevalence of age-related cognitive disorders have led to great interest in studying normal and pathological aging with the aim to individuate early predictors of degenerative disorders, differential diagnosis, and efficacies of pharmacological and cognitive approaches in the treatment of these disorders. Indeed, considering the great burden of degenerative diseases on national healthcare systems in terms of cost and therapies, research aimed at improving the early and differential diagnosis of these pathologies is mandatory.

Alzheimer's Disease (AD) is the first most common neurodegenerative disease affecting millions of people worldwide (Martin et al., 2012). Determination of sensitive and specific markers of very early AD progression is intended to aid researchers and clinicians to develop new treatments and monitor their effectiveness, as well as to lessen the time and cost of clinical trials. To date, individual diagnosis of AD is predominantly based on the clinical examination and neuropsychological assessment (Knopman et al., 2001; Blennow et al., 2006), but definite diagnosis can only be performed by post-mortem analysis.

In the 1980s, the National Institute of Neurologic and Communicative Disorders and Stroke and the Alzheimer's Disease and Related Disorders Association (NINCDS-ADRDA) developed clinical diagnostic criteria for AD by applying a binary approach to diagnosis. According to this approach, a cognitive impairment is necessary for the diagnosis of AD, with definite, probable and possible categories (McKhann et al., 1984). Neuropathological data based on senile plaques and neurofibrillary tangles were afterwards introduced (Hyman and Trojanowski, 1997).

In 2011, revised diagnostic criteria for AD have been developed by the National Institute on Aging-Alzheimer's Association workgroup. These revised diagnostic criteria have replaced the binary approach for a more biological definition of AD: additional supportive features can be obtained by neurogenetic testing, measurement of cerebrospinal fluid (CSF), amyloid and tau, and by neuronal injury biomarkers as measured by neuroimaging studies, including Positron Emission Tomography (PET) and Magnetic Resonance Imaging (MRI). PET and MR changes provide measurements of metabolism/amyloid markers (Fox and Schott, 2004; Jagust et al., 2006) and of atrophic regions, respectively, in order to identify AD, even before dementia is apparent (Albert et al., 2011; Sperling et al., 2011).

Due to the non- invasiveness of MR modality, a considerable effort has been put into the development of advanced MR image processing techniques in order to identify MR-related biomarkers which could be used for enhancing the accuracy of clinical diagnosis of AD. Most studies which were focused on the identification of MR image differences between patients with a clinical diagnosis of AD and healthy subjects were based on a priori-defined regions of interest or on mass univariate image analysis methods (e.g., Voxel Based Morphometry, Busatto et al., 2003; Karas et al., 2003; Ishii et al., 2005). However, both approaches are not able to detect spatially distributed pattern of brain anatomy.

In order to overcome these limitations, in the last few years, there has been a growing interest within the neuroimaging community toward alternative approaches to the analyses of neuroimaging data by considering multivariate pattern analysis, including machine-learning algorithms. Due to their multivariate properties, machine-learning techniques are able to automatically extract multiple information from image sets without requiring a priori hypotheses of where this information may be coded in the images. Several studies have assessed the diagnostic value of these techniques in the classification of AD by cerebral MRI studies (Davatzikos et al., 2008; Klöppel et al., 2008; Gerardin et al., 2009; Cuingnet et al., 2011; Hidalgo-Muñoz et al., 2014), showing promising results also for the prediction of conversion in the early stages of disease (Tufail et al., 2012; Moradi et al., 2015). Among these studies, Klöppel et al. (2008) used machine learning classification and structural MR images for the extraction of spatially-distributed multivariate diagnostic biomarkers. Specifically, the authors were able to identify MR-related biomarkers useful for the differential diagnosis of AD with respect to Fronto-Temporal Lobar Degeneration and normality.

However, early diagnosis of AD by structural MR imaging studies is currently an open challenge due to the difficulty of quantifying patterns of structural change during early stages of AD or during clinically normal stages (Davatzikos et al., 2008). Patients suffering from AD at a prodromal stage are often clinically classified as Mild Cognitive Impairment (MCI), but not all MCI patients convert into AD. A meta-analysis of research and clinical reports suggests that the rate of conversion of MCI to AD is around 5–10% per year (Mitchell and Shiri-Feshki, 2009). Criteria for MCI have been developed (Albert et al., 2011) and various forms have been described (Petersen et al., 1999). Detecting the transition from the asymptomatic phase to symptomatic pre-dementia phase or from the symptomatic pre-dementia phase to dementia onset in the clinical setting is a non-trivial issue (Albert et al., 2011). This causes a diagnostic uncertainty for the early stage of disease.

For this objective, it seems crucial to identify multivariate MR-related diagnostic biomarkers that are able to accurately diagnose MCI converter (MCIc) and MCI non converter (MCInc) with respect to AD and normality. Therefore, different morphological characteristics between normal aging and MCI may be identified by a specific and sensitive analysis of MR images, by revealing which are the most informative image features supporting an early diagnosis (Davatzikos et al., 2008).

In this work we propose a machine learning method able to extract spatially distributed multivariate diagnostic biomarkers from structural MR brain images to be used for the early and accurate diagnosis of AD. In particular, our method is able to identify MRI-related biomarkers of MCI subjects which will convert into AD, opening new prospective in the early management of AD patients.

## Materials and methods

### Participants

Subjects included in this study were obtained from the Alzheimer's Disease Neuroimaging Initiative (ADNI) database (adni.loni.usc.edu). We enrolled 162 cognitively normal elderly controls (CN), 137 patients with diagnosis of AD, 76 patients with diagnosis of MCI who converted to AD within 18 months (MCIc) and 134 patients with diagnosis of MCI who did not convert to AD within 18 months (MCInc). MCI patients who had been followed less than 18 months were not considered. Demographic and clinical data (sex, age and mini-mental score) for each group are shown in Table 1 (see http://adni.loni.usc.edu/study-design/background-rationale/ for further description of groups). A total of 509 subjects from 41 different radiology centers were considered. Identification Numbers (IDs) of each subject involved in this study are reported in Supplementary Tables S1–S4. The ADNI was launched in 2003 by the National Institute on Aging (NIA), the National Institute of Biomedical Imaging and Bioengineering (NIBIB), the Food and Drug Administration (FDA), private pharmaceutical companies and non-profit organizations, as a $60 million, 5-year public private partnership. The primary goal of ADNI has been to test whether serial MR, PET, other biological markers, and clinical and neuropsychological assessment can be combined to measure the progression of MCI and early AD.

**Table 1:** **Demographic and clinical data for the considered groups of participants**.

**Group type**	**# Subjects**	**Age mean ± std [range]**	**Gender # Males/# Females**	**MMSE score mean ± std [range]**	**# Centers**
CN	162	76.3 ± 5.4 [60–90]	76 M/86 F	29.2 ± 1.0 [25–30]	40
MCInc	134	74.5 ± 7.2 [58–88]	84 M/50 F	27.2 ± 1.7 [24–30]	36
MCIc	76	74.8 ± 7.4 [55–88]	43 M/33 F	26.5 ± 1.9 [23–30]	30
AD	137	76.0 ±7.3 [55–91]	67 M/70 F	23.2 ± 2.0 [18–27]	39

According to the ADNI inclusion criteria, enrolled subjects were all between 55 and 90 years of age and spoke either English or Spanish. Each subject was willing, able to perform all test procedures described in the protocol and had a study partner able to provide an independent evaluation of functioning. Inclusion criteria for CN were: Mini Mental State Examination (MMSE) scores between 24 and 30; Clinical Dementia Rating (CDR) (Morris, 1993) of zero; absence of depression, MCI and dementia. Inclusion criteria for MCI were: MMSE scores between 24 and 30; CDR of 0.5; objective memory loss, measured by education adjusted scores on Wechsler Memory Scale Logical Memory II (Wechsler, 1987), absence of significant levels of impairment in other cognitive domains; absence of dementia. Inclusion criteria for AD were: MMSE scores between 20 and 26; CDR of 0.5 or 1.0; NINCDS/ADRDA criteria for probable AD (McKhann et al., 1984; Dubois et al., 2007). Detailed description of inclusion/exclusion criteria can be found in the ADNI protocol (http://www.adni-info.org/Scientists/ADNIStudyProcedures.aspx).

### MR images

T1-weighted structural MR images of all selected subjects were obtained from the ADNI dataset. In order to allow standardization of images from different sites and platforms, we only used images which had undergone: (1) geometry correction for gradient nonlinearity, by 3D gradwarp correction (Jovicich et al., 2006); and (2) intensity correction for non-uniformity, by B1 non-uniformity correction (Narayana et al., 1988). T1-weighted structural MR images of each subject were acquired according to the ADNI acquisition protocol (Jack et al., 2008). MR imaging examinations were performed at 1.5 T. Scans from the baseline visit (when available) or from the screening visit. According to the ADNI protocol, MR imaging examination was performed twice per visit. Scans were then rated by the ADNI investigators of the ADNI MR imaging quality control center at the Mayo Clinic on the basis of blurring/ghosting, flow artifact, intensity, and homogeneity, signal-to-noise ratio (SNR), susceptibility artifacts, and gray-white/cerebrospinal fluid contrast (Jack et al., 2008). In this work, we used the image which was rated as the *best quality scan* for each subject. 3D MR images were downloaded from the ADNI dataset in 3D NIfTI format.

A pre-processing procedure, which mainly aimed at the spatial normalization of all MR images by co-registration to a standard template was applied. All pre-processing procedures were applied to MR images by means of the VBM8 software package (Ashburner and Friston, 2000). First steps of pre-processing consisted in: (1) image re-orientation; (2) cropping; (3) skull-stripping; (4) image normalization to MNI standard space, which was performed by co-registration to the MNI template (MNI152 T1 1 mm brain) (Grabner et al., 2006; O'Hanlon et al., 2013). After co-registration to the MNI template, MR images had a size of 121 × 145 × 121 voxels. Each image was then segmented into Gray Matter (GM) and White Matter (WM) tissue probability maps. Resulting images (whole-brain, GM and WM) were smoothed using an isotropic Gaussian kernel with Full Width at Half Maximum (FWHM) ranging from 2 to 12 mm^3^ (step: 2 mm^3^).

### The classifier

In order to classify the different groups of subjects by means of their T1-weighted structural (whole-brain, GM and WM) we used a machine learning classifier previously implemented by our group (Salvatore et al., 2014). The whole process consists of 2 steps: (1) feature extraction and selection from the MR images of the subjects, which aimed at the selection of the most discriminative features by Principal Components Analysis (PCA) coupled with a Fisher Discriminant Ratio (FDR) criterion (López et al., 2011), and (2) single-subject classification, which aimed at the classification of the subjects on the basis of a predictive model generated for the separation of the different subject groups by means of the most discriminative features (Klöppel et al., 2008; Salvatore et al., 2014).

#### Feature extraction and selection

In order to identify the most discriminative features among groups, an automatic feature extraction technique was applied to MR images (whole-brain, GM and WM). This technique also allowed to reduce the number of features to be handled without losing relevant information for discrimination, and thus to enhance computational performances of the machine learning algorithm.

PCA was implemented to perform feature extraction (Habeck et al., 2008; López et al., 2011). This technique is based on two consecutive steps: (1) application of an orthogonal transformation to a dataset of (possibly) correlated variables; this operation results in a set of values of orthogonal (uncorrelated) variables, which are referred to as Principal Components of the original dataset and which define the so-called PCA subspace; (2) projection of each variable of the original dataset onto the PCA subspace; this operation results in the reduction of the original set of observed variables into a smaller set of features, which are referred to as PCA coefficients and which can be used in subsequent analyses. The total number of PCA coefficients is equal to the number of Principal Components extracted from the original dataset.

Mathematically, if we consider a dataset A composed of S samples, with each sample being a collection of N variables, then the dimension of the dataset is S × N. By computing the eigenvectors of the covariance matrix of the dataset A, PCA subspace can be defined as the space spanned by these eigenvectors. Application of PCA to the dataset A results in a number of Principal Components (i.e., of eigenvectors) with non-zero eigenvalues which is at most equal to the value of the smaller dimension of the dataset–1. Principal Components are sorted in descending order according to the proportion of variance explained, with the constraint for them to be orthogonal with each other.

In this study, datasets were composed of S samples (MR images), where the dimension N of each pre-processed MR image was 121 × 145 × 121 voxels. Application of PCA to our datasets resulted in a number of Principal Components with non-zero eigenvalues which was at most equal to the number S of samples in each dataset–1. The dimension of each dataset after application of PCA was S × (S – 1).

PCA coefficients resulting from the feature extraction process were then sorted in a descending order according to their FDR, which gives information about the class discriminatory power of a given component. For each component, FDR can be calculated as follows:

(1) $$\begin{array}{l} FDR=\frac{{\left(\mu_{1}-\mu_{2}\right)}^{2}}{\sigma_{1}^{2}+\sigma_{2}^{2}} \end{array}$$

where μ_*i*_ and $\sigma_{i}^{2}$ are the mean and the variance of the ith class, respectively.

The explained variance was studied as a function of the number of considered principal components before and after sorting them in accordance to their FDR, in order to show the impact of FDR-analysis on PCA coefficients.

#### Classification

The classification algorithm of the proposed machine learning method was based on Support Vector Machines (SVM) (Schölkopf and Smola, 2002). The aim of SVM is to find a predictive model which is able to perform binary group separation. This predictive model is represented by a hyper-plane which can be computed using a set of data input to SVM for its training (training data). The set of training data consists of: (1) a vector of samples belonging to two different classes and (2) the corresponding vector of labels (two labels, each label identifies one class). SVM is able to compute a predictive model for the classification of a new sample to one or to the other of the two classes. Specifically, the predicted class *y* for the sample *x* is given by the following relation:

(2) $$\begin{array}{l} y\left(x\right)=\overset{N}{\underset{n=1}{\sum }}w_{n}\cdot t_{n}\cdot k\left(x,x_{n}\right)+b \end{array}$$

where *N* is the number of samples included in the training set; *w*_*n*_ is a weight assigned by SVM to each sample *n* in the training set during the training phase; *t*_*n*_ is the label of the sample *n* of the training set; *k(x, x*_*n*_*)* is a kernel function; b is a threshold parameter. The main difference among SVM and other classification methods is that the hyper-plane computed by SVM is the one which maximizes the separation between the two classes.

In this work, we used the Matlab platform to both implement and optimize the SVM classifier. We used a linear kernel for all analyses. Our code also included algorithms of the biolearning toolbox of Matlab.

### Optimization of classification and evaluation of accuracy

An optimization was performed with the purpose of finding the best parameter configuration for the classification of the different groups of subjects. A Nested Cross Validation (Nested CV) was used. In this approach, the original dataset is split into k subsets of (possibly) equal size. An inner training-and-validation loop for parameter estimation and optimization is performed using k-1 subsets, and an outer test loop for performance evaluation is performed using the kth held-out subset. This procedure is then repeated k times, until all k subsets are used once for performance evaluation.

In this study, we performed nested 20-fold CV using 19/20 of the original data for the inner training and validation loop allowing parameter estimation and optimization. For each inner loop, these 19/20 subsets were randomly split in half in order to perform training and validation on two independent datasets. The trained and optimized model and parameter set were then used to predict the held-out 1/20 subset.

For each round, the optimal parameters (which brain tissue, which level of filtering, how many PCA coefficients) were chosen as those for which the classification error (E) was minimized. Specifically, we aimed at minimizing the quantity given by,

(3) $$\begin{array}{rcl} E & = & 1\;-Balanced\;Accuracy \end{array}$$

(4) $$\begin{array}{rcl} Balanced\;Accuracy & = & \;\frac{1}{2}\left(Specificity+Sensitivity\right) \end{array}$$

as a function of the following parameters: (1) tissue map (whole-brain, GM and WM); (2) smoothing (FWHM = 2, 4, 6, 8, 10, 12 mm^3^, or no smoothing); (3) number of PCA coefficients (from 1 to PC, where PC is the total number of extracted coefficients).

For each of the 20 separate rounds of the outer loop, Balanced Accuracy was calculated and results were averaged across all 20 rounds (Overall Balanced Accuracy).

Parameter optimization and accuracy evaluation were performed for the three following comparisons: (1) AD vs. CN, (2) MCIc vs. CN, and (3) MCIc vs. MCInc.

It is worth noting that pre-processing, feature extraction and feature selection steps were performed separately on the datasets used in the inner training-and-validation loop and in the outer test loop, in order to avoid over-fitting problems (Kuncheva, 2004).

### Diagnostic MR-related biomarkers

Extraction of MR-related biomarkers was carried out according to the following procedure. For each round of the inner training-and-validation loop, maps of voxel-based pattern distribution of MR image differences among groups of subjects were generated for the optimized configuration (minimum E), thus obtaining 20 maps. These maps were averaged in order to obtain the final map. This procedure was applied to the three following comparisons: (1) AD vs. CN, (2) MCIc vs. CN, and (3) MCIc vs. MCInc.

The importance of each considered sample for the classification was computed on the basis of the predictive model generated by our SVM (Klöppel et al., 2008; Focke et al., 2011; Salvatore et al., 2014). As specified in Equation (2), the weight *w*_*n*_, assigned by the SVM to the sample *n* during the training phase of the classification, indicates the importance of that sample for the computation of the separating hyper-plane and, thus, indicates the importance of that sample for the separation of the two considered groups. It is worth noting that the weight *w*_*n*_ assigned by SVM to the sample *n* is non-zero only for support vectors, being respectively positive or negative depending on the class to which the sample *n* belongs. Each sample *n* of the training set was multiplied by the corresponding assigned weight *w*_*n*_. Resulting weighted samples were added in order to generate a vector representing the weight of each feature for the classification. In order to ensure the correct interpretation of weights assigned by SVM, we then applied the method proposed by Haufe and colleagues to compute activation patterns for backward models as described in Haufe et al. (2014). The computed pattern was finally transformed back from the PCA space to the MR-images space, resulting in a map of voxel-based pattern distribution of MR image differences among groups.

Voxel-based pattern distribution (normalized to a range between 0 and 1) was represented by a proper color scale and superimposed on a standard stereotactic brain for spatial localization. In this way, MR-related diagnostic biomarkers for the diagnosis of AD (AD vs. CN) and for the early diagnosis of AD (MCIc vs. CN, and MCIc vs. MCInc) were identified.

## Results

### Participants

Groups of participants did not show significant differences for age (Student's *t*-test with significance level at 0.05) and gender (Pearson's chi-square test with significance level at 0.05). Significant differences for MMSE scores were found between CN and patients (AD, MCIc) (Student's *t*-test with *p* < 0.0001), consistently with previous studies considering the same groups of ADNI subjects (Cuingnet et al., 2011).

### MR images

**Figure 1:**
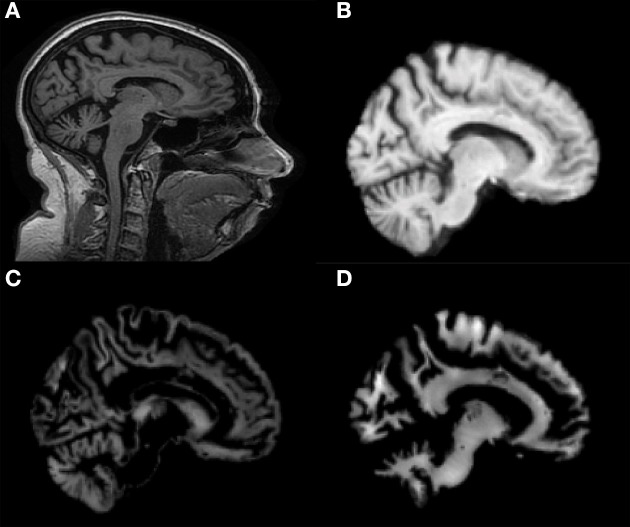
**Sagittal image of a MR scan from a MCIc patient: (A) original image; (B) same slice, deskulled and co-registered to the MNI space; same slice, segmented into Gray Matter (GM) (C) and into White Matter (WM) (D)**.

Co-registration of all MRI images to the MNI template and segmentation into GM and WM tissue probability maps were performed correctly. Figure 1 shows results of these procedures for a representative MR image of a MCIc patient. Sagittal view of the original volume (A), the slice co-registered to the MNI space (B), the GM tissue probability map (C) and the WM tissue probability map (D) are shown.

### The classifier

#### Feature extraction and selection

**Figure 2:**
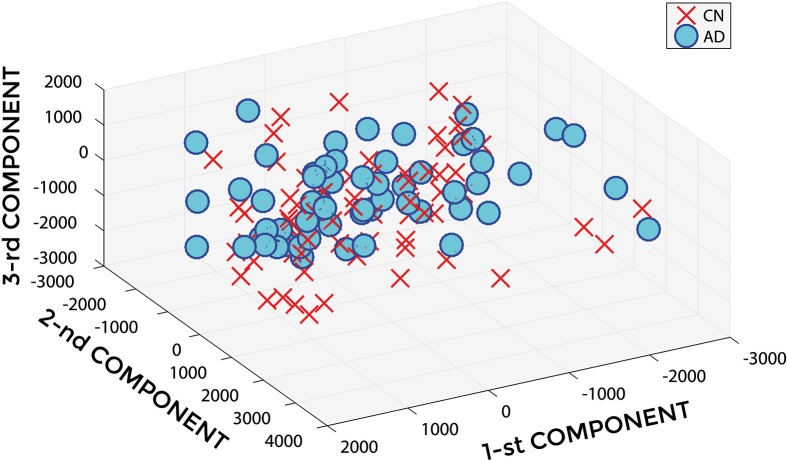
**PCA coefficients for the comparison between AD (o symbol) and CN (× symbol) when using GM tissue probability map and an isotropic Gaussian kernel with 10 mm^3^ FWHM for smoothing**. 1st, 2nd, and 3rd components are shown.

Figure 2 shows a representative example of PCA coefficients resulting from the feature extraction and selection obtained from the comparison between AD and CN. 1st, 2nd, and 3rd components are shown when using GM tissue probability map and an isotropic Gaussian kernel with 10 mm^3^ FWHM for smoothing. The number of the extracted PC was 141.

**Figure 3:**
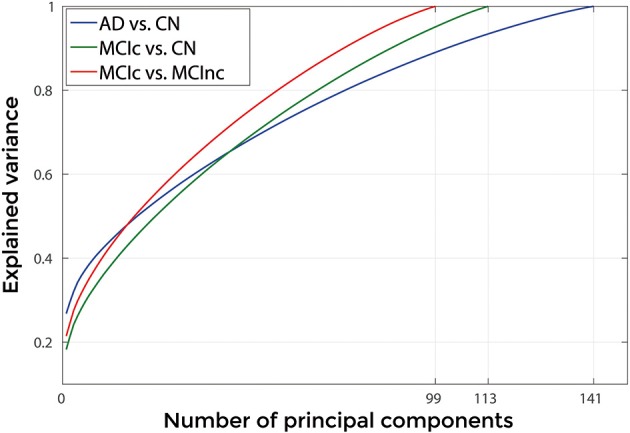
**Explained Variance as a function of the number of considered Principal Components, when using GM tissue probability map and no smoothing, for the following comparisons: AD vs. CN, MCIc vs. CN, MCIc vs. MCInc**.

**Figure 4:**
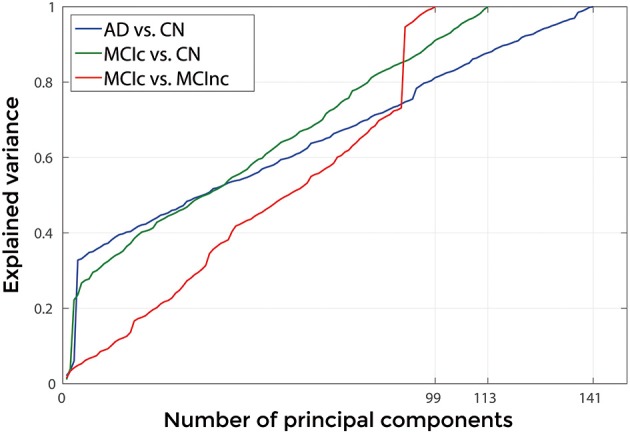
**Explained Variance as a function of the number of considered principal components sorted in accordance to their FDR, when using GM tissue probability map and no smoothing, for the following comparisons: AD vs. CN, MCIc vs. CN, MCIc vs. MCInc**.

Figures 3, 4 show, as representative examples, the explained variance as a function of the number of considered PCs, before (Figure 3) and after (Figure 4) sorting them in accordance to their FDR. Plots are shown for the comparisons between AD and CN, MCIc, and CN, MCIc, and MCInc when using GM tissue probability maps and no smoothing. The trend of explained variance as a function of the number of considered PCs was modified by the application of FDR-analysis. In particular, FDR-analysis allowed the most discriminative information for class separation to be contained in the first few principal components. This is shown by the step in the explained variance in correspondence with a low number of components for the comparisons between AD vs. CN and MCIc vs. CN (Figure 4).

#### Classification

**Figure 5:**
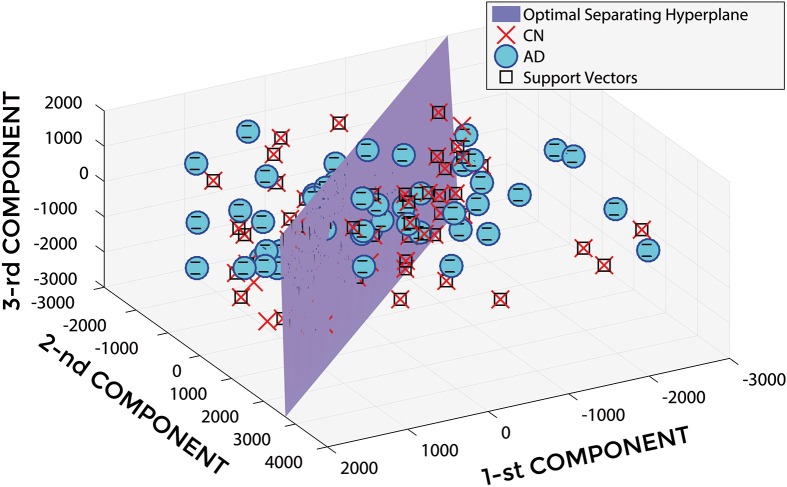
**Hyper-plane plane separating AD (o symbol) from CN (× symbol) PCA coefficients (3 PCA coefficients), and defined Support Vectors (□ symbol), when using GM tissue probability map and an isotropic Gaussian kernel with 10 mm^3^ FWHM for smoothing**. 1st, 2nd, and 3rd components are shown.

In Figure 5, a representative example of the hyper-plane separating AD from CN subjects is shown when using 3 PCA coefficients, GM tissue probability map and an isotropic Gaussian kernel with 10 mm^3^ FWHM for smoothing. The number of subjects involved was 142 (67 AD, 75 CN) and the total number of extracted PCA coefficients was 141.

### Optimization of classification and evaluation of accuracy

**Figure 6:**
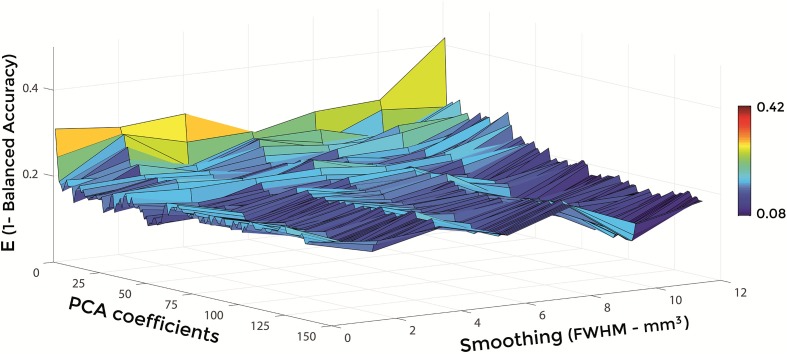
**E (1 – Balanced Accuracy) as a function of smoothing (FWHM – mm^3^) and number of PCA coefficients for the comparison between AD and CN when using GM**.

**Figure 7:**
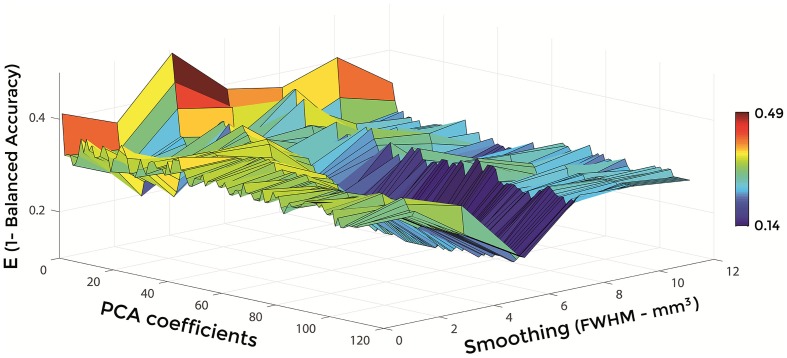
**E (1 – Balanced Accuracy) as a function of smoothing (FWHM – mm^3^) and number of PCA coefficients for the comparison between MCIc and CN when using GM**.

**Figure 8:**
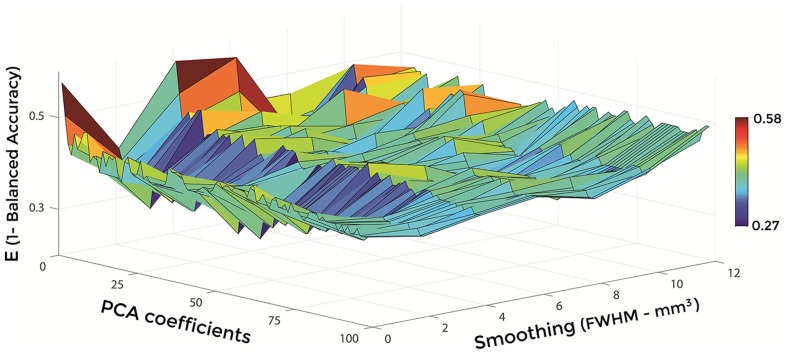
**E (1 – Balanced Accuracy) as a function of smoothing (FWHM – mm^3^) and number of PCA coefficients for the comparison between MCIc and MCInc when using GM**.

Figures 6–8 show E (1 – Balanced Accuracy) as a function of applied smoothing (FWHM – mm^3^) and number of PCA coefficients when using GM tissue probability maps. Plots are shown for the comparisons between AD and CN, MCIc, and CN, MCIc, and MCInc.

Optimal parameters resulting from classifier optimization are reported in Table 2. For all the comparisons, minimum values of E were obtained mostly when using GM tissue probability maps (frequency of 100% for AD vs. CN, 85% for MCIc vs. CN, 80% for MCIc vs. MCInc). For the comparison between AD and CN, the best set of optimal parameters among the 20 rounds was: GM tissue probability map; 10 mm^3^ FWHM of the isotropic Gaussian kernel for smoothing; 127 PCA coefficients. When using these parameters, E reached its minimum value of 0.08. For the comparison between MCIc and CN, the best set of optimal parameters among the 20 rounds was: GM tissue probability map; 6 mm^3^ FWHM of the isotropic Gaussian kernel for smoothing; 67 PCA coefficients. When using these parameters, E reached its minimum value of 0.14. For the comparison between MCIc and MCInc, the best set of optimal parameters among the 20 rounds was: GM tissue probability map; 2 mm^3^ FWHM of the isotropic Gaussian kernel for smoothing; 34 PCA coefficients. When using these parameters, E reached its minimum value of 0.27.

**Table 2:** **Classification error and optimal parameters (Tissue map, Smoothing, Number of PCA coefficients) for each of the 20 rounds of the inner training-and-validation loop (best configuration in bold)**.

**Comparison**	**E**	**Tissue map**	**Smoothing FWHM [mm^3^]**	**PCA coefficients**
AD vs. CN	0.10 **0.08** 0.12 0.11 0.11 0.15 0.13 0.12 0.12 0.11 0.12 0.09 0.13 0.12 0.12 0.13 0.12 0.15 0.11 0.12	GM **GM** GM GM GM GM GM GM GM GM GM GM GM GM GM GM GM GM GM GM	6 **10** 10 4 6 2 8 4 2 2 4 4 8 2 4 2 2 6 6 12	6 **127** 41 62 75 64 69 32 67 50 48 54 35 118 46 22 135 49 54 30
MCIc vs. CN	0.19 0.17 0.20 0.19 0.22 0.20 0.15 0.15 0.21 0.19 **0.14** 0.19 0.19 0.17 0.22 0.18 0.19 0.19 0.19 0.19	GM GM GM GM WB WB GM GM GM GM **GM** GM WB GM GM GM GM GM GM GM	8 2 2 4 2 10 4 10 10 10 **6** 4 6 10 8 12 12 10 8 8	26 25 94 53 14 57 62 22 75 32 **67** 13 64 80 28 21 16 81 76 101
MCIc vs. MCInc	0.30 0.31 0.33 0.34 0.32 0.30 0.33 0.28 **0.27** 0.31 0.31 0.32 0.32 0.30 0.34 0.33 0.32 0.28 0.30 0.30	GM GM WB GM GM GM WB GM **GM** WM GM GM GM GM GM GM WB GM GM GM	2 10 12 4 8 6 2 6 **2** 4 2 8 8 4 8 8 4 10 8 2	9 19 34 34 16 17 21 10 **34** 4 16 31 23 46 33 2 34 5 8 84

The operational time required by the whole pre-processing and training of the classifier (including feature extraction and selection) using the best set of optimal parameters, as measured by the *tic* and *toc* functions implemented in Matlab (version R2015a) and running on a system with 32 CPUs at 2.00 GHz, was 31.7s for the comparison between AD and CN, 21.7s for the comparison between MCIc and CN and 21.2s for the comparison between MCIc and MCInc. The testing phase, including preprocessing and classification of the new dataset, took 1.5s per subject on average.

The Overall Balanced Accuracy (averaged across all 20 rounds) was 0.76 ± 0.11 for the classification of AD vs. CN, 0.72 ± 0.12 for the classification of MCIc vs. CN, 0.66 ± 0.16 for the classification of MCIc vs. MCInc, respectively.

Since MMSE resulted significantly different between CN and patients (AD, MCIc), we have also tested our classification algorithm after incorporating MMSE as additional feature. Balanced Accuracy resulted to be affected (from 0.76 ± 0.11 to 0.99 ± 0.03 for AD vs. CN, from 0.72 ± 0.12 to 0.78 ± 0.16 for MCIc vs. CN, from 0.66 ± 0.16 to 0.60 ± 0.17 for MCIc vs. MCInc).

**Figure 9:**
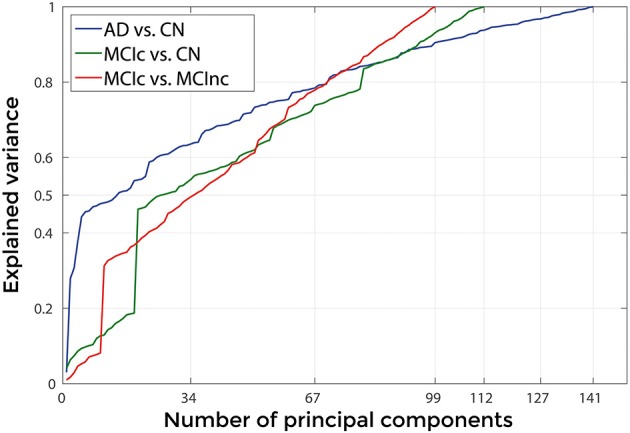
**Explained Variance, when using the best set of optimal parameters, as a function of the number of considered principal components sorted in accordance to their FDR, for the following comparisons: AD vs. CN, MCIc vs. CN, MCIc vs. MCInc**.

Figure 9 shows the explained variance as a function of the number of considered PC sorted in accordance to their FDR. Plots are shown for the comparisons between AD and CN, MCIc and CN, MCIc and MCInc when using the best configuration highlighted in Table 2. For AD vs. CN comparison, the percentage of variance explained by the first 127 components was 98%; for MCIc vs. CN comparison, the percentage of variance explained by the first 67 components was 74%; for MCIc vs. MCInc comparison, the percentage of variance explained by the first 34 components was 50%.

### Diagnostic MR-related biomarkers

**Figure 10:**
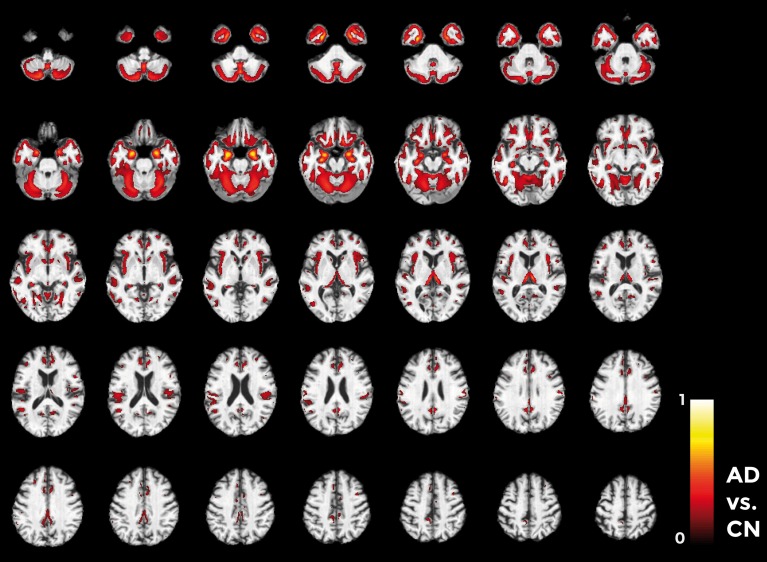
**Voxel-based pattern distribution map (axial view) for the classification between AD and CN**. Voxel-based pattern distribution (normalized to a range between 0 and 1) is expressed according to the color scale (threshold = 50%) and superimposed on a standard stereotactic brain for spatial localization.

**Figure 11:**
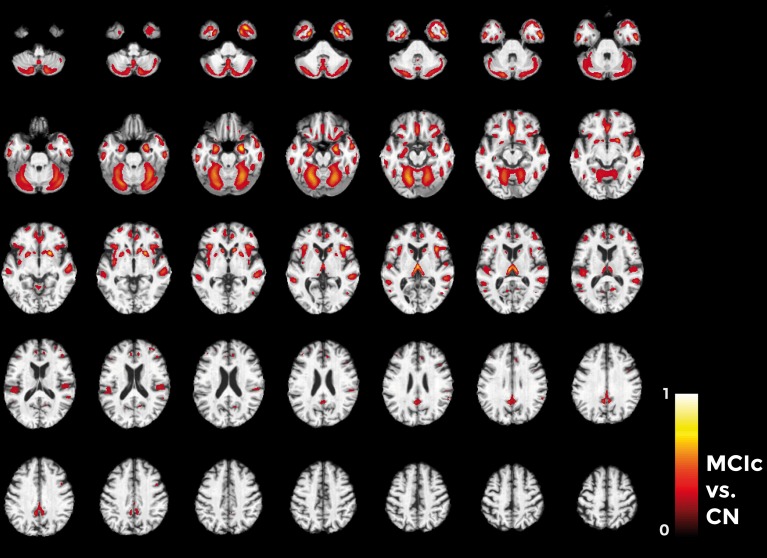
**Voxel-based pattern distribution map (axial view) for the classification between MCIc and CN**. Voxel-based pattern distribution (normalized to a range between 0 and 1) is expressed according to the color scale (threshold = 45%) and superimposed on a standard stereotactic brain for spatial localization.

**Figure 12:**
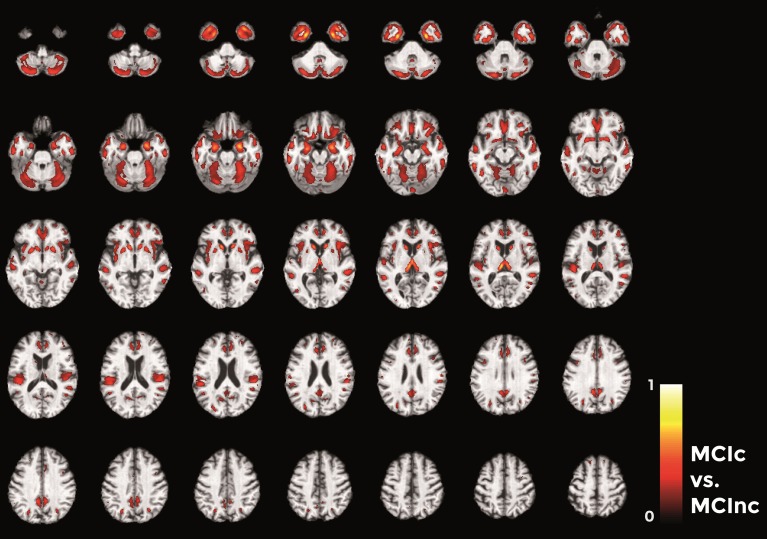
**Voxel-based pattern distribution map (axial view) for the classification between MCIc and MCInc**. Voxel-based pattern distribution (normalized to a range between 0 and 1) is expressed according to the color scale (threshold = 35%) and superimposed on a standard stereotactic brain for spatial localization.

Figures 10–**12** show voxel-based pattern distribution maps for the three following classification: (1) AD vs. CN, (2) MCIc vs. CN, (3) MCIc vs. MCInc. The pattern of differences (normalized to a range between 0 and 1) is expressed according to the color scales.

Voxels influencing the classification of AD with respect to CN (Figure 10) are localized in the temporal pole, superior temporal cortex, medial temporal cortex including hippocampus and entorhinal cortex, amygdala, thalamus, putamen, caudate, insular cortex, gyrus rectus, lateral orbitofrontal cortex, inferior frontal cortex, superior frontal cortex, anterior cingulate cortex, precuneus, and in the posterior cerebellar lobule.

Considering the comparison between MCIc and CN individuals (Figure 11), the major part of voxel-based pattern distribution was similar to the one previously found in AD.

Finally, in the direct comparisons between the two MCI groups (Figure 12) we detected only voxels influencing classification of the MCIc with respect to MCInc. In other words, there were no anatomical changes in the MCInc's brain useful to increase the accuracy of classification. Overall, the major part of voxel-based pattern distribution was similar to the one detected in the previous MCIc vs. CN contrast.

## Discussion and conclusions

The localization and spatial extent of the anatomical features identified in our study are in line with previous research showing the precedence of pathologic changes in the temporal and parietal cortex (Braak and Braak, 1991; Schroeter et al., 2009). In fact, a recent neuroimaging meta-analysis (Schroeter et al., 2009) aimed at characterizing the prototypical neural substrates of AD and its prodromal stage amnestic MCI reported the presence of:

- (a) Reduction of glucose utilization and perfusion in the inferior parietal lobules and the posterior cingulate cortex and precuneus; hypometabolism was detected in the left anterior superior insula; whereas gray matter atrophy was found in the left temporal pole/anterior superior temporal sulcus, right amygdala, and gyrus rectus when 525 MCI patients were compared with 1097 healthy controls.
- (b) Reductions in glucose utilization and perfusion coincided in the inferior parietal lobules, posterior superior temporal sulcus, precuneus, posterior cingulate cortex, anterior medial frontal cortex, anterior cingulate gyrus and right inferior temporal sulcus; hypometabolism was in the right frontal pole, left posterior middle frontal gyrus and left hippocampal head; whereas gray matter atrophy was found in the both amygdalae, both anterior hippocampal formations, entorhinal areas, medial thalamus, posterior insula, and left middle temporal gyrus/superior temporal sulcus when 826 AD patients were compared with 1097 healthy controls.

The only brain region revealed by our pattern recognition analysis not typically related to AD-like atrophy was the cerebellum. The cerebellum is a region generally rather neglected in AD research. Atrophy of this region has been sparsely reported in neuroimaging studies (Thomann et al., 2008a; Nigro et al., 2014), although there is considerable number of histo-pathological studies that demonstrated the presence of degenerative changes (Li et al., 1999; Wegiel et al., 2000; Wang et al., 2002). These alterations mainly comprise reduced Purkinje cell density, atrophy of the molecular and granular cell layer as well as a large number of amyloid plaques in the cerebellar cortex of AD compared to controls. Moreover the fact that we detected only anatomical changes in the posterior lobule of the cerebellum corroborated our findings, since cognitive performance in AD patients was found to be significantly correlated with volumes of posterior cerebellar lobes (Thomann et al., 2008b).

The development of computer-based automatic methods for the accurate classification of patients in early phase of AD from imaging data has attracted strong interest from the clinical community in the last few years, since its possible critical impact on clinical management and practice (i.e., identification of new biomarkers). Many of these classification methods are based on SVM, a set of algorithms that uses supervised learning of pattern recognition in a training set to build a classifier able to predict the category to which a new example belongs. One of the most important challenging in this field of study is to define automated methods to discriminate MCI patients progressing later to AD from patients who will not (Schroeter et al., 2009). For this reason, this study was aimed at assessing the powerful of machine learning methods in discriminating MCI at a risk state of AD.

In our work we used nested CV to measure the performance of our classifier. Nested CV avoids optimistically biased estimates of performance that may arise from the use of the same CV for parameter estimation and performance evaluation. Specifically, when model parameters are estimated by means of the performance evaluation criterion, then these estimates depend on (1) improvements in generalization performance and (2) statistical features of the particular dataset on which the performance are evaluated. This may result in under-estimates of the CV error. Moreover, in ordinary CV, parameter estimation is performed prior to model building, which could lead to an optimistic evaluation of the performance of the classifier. On the other side, in nested CV parameter estimation is performed simultaneously to performance evaluation (Cawley and Talbot, 2010).

Performances of our classification algorithm evaluated by nested 20-fold CV were 0.76 for AD vs. CN, 0.72 for MCIc vs. CN, and 0.66 for MCIc vs. MCInc. In their published study, Cuingnet et al. (2011) evaluated the performance of ten different machine learning methods (28 algorithm configurations) by using the same group of ADNI subjects employed in our work, splitting datasets in two equal sample groups and using one group to estimate the optimal value of hyperparameters and the other group to evaluate the performance of the classifier. Performances reached by our algorithm for the three classifications (AD vs. CN, MCIc vs. CN, and MCIc vs. MCInc) are better than 27/28 algorithm configurations, since 27 algorithms have a Balanced Accuracy lower than 0.66 for the MCIc vs. MCInc comparison.

The use of our classifier is limited to the early diagnosis of AD. Notwithstanding the vast majority of brain regions identified by our multivariate pattern recognition analysis have been also described to be involved in neurodegenerative processes underlying other dementia disorders (e.g., Fronto-Temporal Lobar Degeneration), machine learning has been also found accurate when applied to MR images for the differential diagnosis of AD (e.g., Klöppel et al., 2008). The clinical use of such a machine learning approach (early and differential diagnosis of AD) should require the training of a multicategory classifier (Beom Choi et al., 2014) on MR images from CN and different dementia patients (e.g., MCIc, MCInc, AD, FTD).

The main innovative result of our work was the extraction of MR-related biomarkers for the early diagnosis of AD by means of machine learning. We assessed the relevance of each brain voxel with respect to the classification analysis, thus allowing regions critically involved in the pathophysiological mechanisms of AD to be identified. Notably, the vast majority of brain regions allowing to perform the best discrimination between AD and CN, as well as between MCIc and CN, were the same regions allowing the discrimination between the two critical forms of MCI, i.e., MCIc and MCInc. In other words, the AD-like atrophy patterns characterized by combined pathological changes within the temporal cortex, hippocampus, entorhinal cortex, thalamus, insular cortex, anterior cingulate cortex, orbitofrontal cortex, and precuneus, allowed distinguishing clinically- and cognitively-matched MCI patients progressing to AD from those who will not.

In conclusion, we demonstrated that an advanced neuroimaging approach based on machine learning is able to accurately classify patients who will or will not develop AD by means of structural MRI data and to extract MR-related biomarkers of AD. Moreover, our advanced neuroimaging study allows us to perform a challenging reflection. Due to the similarity between AD-like atrophy patterns with those detected in MCI who will convert in AD, we can derive that the machine learning approach impacts on the sensitivity of AD-related features rather than specificity. This would suggest that the problem of how to perform diagnosis of AD at a very early stage by MRI seems to be a matter of increasing the MRI detectability of structural biomarkers. For this reason, both current generation MRI systems combined with advanced images processing algorithms and future generation MRI systems with improved sensitivity (e.g., increased MRI resolution and better S/N ratio) will –definitely– move MRI diagnostic role from clinical to pre-clinical stage of AD.

### Conflict of interest statement

The authors declare that the research was conducted in the absence of any commercial or financial relationships that could be construed as a potential conflict of interest.
