# Supplementary material for: Magnetic resonance imaging biomarkers for the early diagnosis of Alzheimer's disease: a machine learning approach
Source: Front Neurosci. 2015 Sep 1;9:307. doi: 10.3389/fnins.2015.00307 (PMC4555016; doi:10.3389/fnins.2015.00307)
Supplement: Supplementary file 1 [file Table1.DOCX]

***Supplementary Material***

**Magnetic Resonance Imaging biomarkers for the early diagnosis of Alzheimer’s Disease: a machine learning approach**

**Christian Salvatore^1^, Antonio Cerasa^2^, Petronilla Battista^1^, Maria Carla Gilardi^1^, Aldo Quattrone^3^, Isabella Castiglioni^1*^ and the Alzheimer’s Disease Neuroimaging Initiative^§^**

^1^Institute of Molecular Bioimaging and Physiology, National research Council (IBFM-CNR), Segrate, Milan, Italy

^2^Neuroimaging Research Unit, Institute of Molecular Bioimaging and Physiology, National Research Council (IBFM-CNR), Catanzaro, Italy

^3^Institute of Neurology, Department of Medical Sciences, University “Magna Graecia”, Catanzaro, Italy

**^§^** Data used in preparation of this article were obtained from the Alzheimer’s Disease Neuroimaging Initiative (ADNI) database (adni.loni.usc.edu). As such, the investigators within the ADNI contributed to the design and implementation of ADNI and/or provided data but did not participate in analysis or writing of this report. A complete listing of ADNI investigators can be found at: <http://adni.loni.usc.edu/wp-content/uploads/how_to_apply/ADNI_Acknowledgement_List.pdf>

*** Correspondence:** Isabella Castiglioni, Institute of Molecular Bioimaging and Physiology, National research Council (IBFM-CNR), Via F.lli Cervi, 93, Segrate, Milan, Italy. isabella.castiglioni@ibfm.cnr.it

1. **Supplementary Tables**

**Supplementary Table 1.** Identification data (Center ID, Subject ID, SID, UID) of CN subjects involved in this study.

| **CN DATASET** | | | | | | | |
| --- | --- | --- | --- | --- | --- | --- | --- |
| **CENTER ID** | **SUBJECT ID** | **SID** | **UID** | **CENTER ID** | **SUBJECT ID** | **SID** | **UID** |
| 2 | 295 | 13408 | 45109 | 36 | 1023 | 24338 | 65105 |
| 2 | 1261 | 26574 | 62378 | 41 | 1002 | 23705 | 65221 |
| 2 | 1280 | 26453 | 60057 | 57 | 643 | 15782 | 34726 |
| 3 | 907 | 19728 | 52782 | 57 | 779 | 18109 | 80630 |
| 3 | 981 | 20753 | 52777 | 57 | 934 | 19971 | 34735 |
| 5 | 602 | 15966 | 32674 | 67 | 19 | 9539 | 45229 |
| 6 | 484 | 18837 | 65567 | 73 | 312 | 15079 | 39888 |
| 6 | 498 | 15857 | 67058 | 73 | 386 | 13746 | 49681 |
| 6 | 681 | 18451 | 92306 | 82 | 363 | 13017 | 39731 |
| 7 | 68 | 10356 | 35791 | 82 | 640 | 16562 | 49480 |
| 7 | 1222 | 25402 | 60004 | 94 | 489 | 14121 | 47292 |
| 11 | 2 | 9107 | 35470 | 94 | 711 | 16553 | 39139 |
| 11 | 5 | 9136 | 32247 | 98 | 171 | 11460 | 65753 |
| 11 | 16 | 9253 | 32307 | 98 | 896 | 19439 | 56032 |
| 11 | 21 | 9581 | 32342 | 99 | 533 | 14938 | 38786 |
| 11 | 22 | 9617 | 32394 | 109 | 1014 | 24818 | 63480 |
| 11 | 23 | 8868 | 32410 | 114 | 173 | 11594 | 96322 |
| 13 | 502 | 17232 | 51139 | 114 | 601 | 15201 | 39851 |
| 13 | 575 | 17859 | 51161 | 126 | 405 | 14635 | 38828 |
| 13 | 1035 | 21984 | 51166 | 126 | 506 | 15652 | 38855 |
| 14 | 519 | 14488 | 39648 | 126 | 605 | 15665 | 38705 |
| 14 | 520 | 14473 | 39661 | 126 | 680 | 16099 | 38927 |
| 14 | 558 | 15789 | 39684 | 127 | 260 | 12419 | 34385 |
| 20 | 883 | 19459 | 60674 | 127 | 622 | 15473 | 34453 |
| 20 | 899 | 19329 | 60628 | 128 | 863 | 19272 | 98876 |
| 20 | 1288 | 26890 | 60665 | 130 | 232 | 11928 | 39110 |
| 22 | 14 | 9271 | 59376 | 130 | 969 | 20385 | 39277 |
| 22 | 130 | 11089 | 59525 | 130 | 1200 | 25090 | 63753 |
| 23 | 61 | 10312 | 31103 | 131 | 123 | 10960 | 63785 |
| 23 | 963 | 19740 | 89418 | 131 | 319 | 12322 | 47985 |
| 24 | 985 | 21112 | 62716 | 131 | 441 | 13681 | 48030 |
| 24 | 1063 | 21525 | 63393 | 133 | 433 | 13952 | 39947 |
| 27 | 118 | 11796 | 34115 | 133 | 488 | 14150 | 107935 |
| 27 | 403 | 13717 | 34182 | 136 | 86 | 13191 | 66414 |
| 29 | 824 | 18139 | 96285 | 136 | 196 | 13253 | 40261 |
| 29 | 843 | 18909 | 66998 | 941 | 1194 | 25323 | 63848 |
| 33 | 920 | 19288 | 42482 | 941 | 1195 | 26180 | 63866 |
| 33 | 1098 | 22792 | 42833 | 941 | 1197 | 25332 | 66463 |
| 35 | 48 | 10257 | 45184 | 941 | 1202 | 25680 | 63875 |
| 36 | 672 | 17131 | 36939 | 941 | 1203 | 25671 | 63880 |
| 36 | 813 | 18252 | 36980 | 141 | 1094 | 23294 | 47733 |
| 2 | 413 | 13893 | 45118 | 52 | 1250 | 25829 | 62241 |
| 2 | 559 | 14875 | 40675 | 52 | 1251 | 26231 | 62283 |
| 2 | 685 | 16309 | 40684 | 62 | 578 | 15035 | 50460 |
| 3 | 931 | 20051 | 53391 | 62 | 768 | 17527 | 50507 |
| 3 | 1021 | 21771 | 73507 | 62 | 1099 | 22713 | 50558 |
| 5 | 553 | 15527 | 32646 | 67 | 56 | 8723 | 35894 |
| 5 | 610 | 15727 | 32669 | 67 | 59 | 10517 | 35903 |
| 6 | 731 | 18321 | 90849 | 67 | 177 | 12187 | 34807 |
| 7 | 70 | 10950 | 36629 | 67 | 257 | 14730 | 34825 |
| 7 | 1206 | 25173 | 59982 | 73 | 89 | 11161 | 49676 |
| 11 | 8 | 9195 | 32265 | 73 | 311 | 15069 | 39869 |
| 13 | 1276 | 27641 | 62681 | 82 | 304 | 12557 | 95654 |
| 14 | 548 | 14921 | 39666 | 82 | 761 | 18119 | 39792 |
| 16 | 359 | 13000 | 96222 | 82 | 1256 | 26812 | 63157 |
| 16 | 538 | 17545 | 40773 | 94 | 526 | 14559 | 63469 |
| 20 | 97 | 10858 | 64047 | 94 | 692 | 17207 | 47296 |
| 22 | 66 | 10271 | 59447 | 94 | 1267 | 28217 | 80719 |
| 22 | 96 | 11006 | 59457 | 98 | 172 | 11812 | 65758 |
| 23 | 31 | 9785 | 69613 | 99 | 40 | 8920 | 34608 |
| 23 | 58 | 10335 | 30551 | 99 | 90 | 10835 | 35842 |
| 23 | 81 | 10813 | 31126 | 99 | 352 | 12992 | 34538 |
| 23 | 926 | 19390 | 31548 | 99 | 534 | 14009 | 34579 |
| 23 | 1190 | 24847 | 46418 | 109 | 876 | 19132 | 82595 |
| 23 | 1306 | 26604 | 46436 | 109 | 967 | 21137 | 55045 |
| 27 | 74 | 10605 | 34317 | 109 | 1013 | 22585 | 66150 |
| 27 | 120 | 11535 | 34333 | 114 | 166 | 11584 | 39811 |
| 29 | 845 | 18829 | 64868 | 114 | 416 | 13556 | 39837 |
| 29 | 866 | 18917 | 65612 | 127 | 259 | 12137 | 34363 |
| 33 | 516 | 14818 | 42309 | 127 | 684 | 16759 | 34459 |
| 33 | 734 | 16942 | 42435 | 130 | 886 | 19561 | 39173 |
| 33 | 741 | 17006 | 42451 | 131 | 436 | 14710 | 48021 |
| 33 | 923 | 19544 | 42510 | 131 | 1301 | 26899 | 63794 |
| 33 | 1016 | 21817 | 42773 | 133 | 493 | 14156 | 107944 |
| 33 | 1086 | 23547 | 42786 | 133 | 525 | 14991 | 39981 |
| 35 | 156 | 11391 | 39534 | 136 | 184 | 11974 | 40180 |
| 35 | 555 | 15332 | 39602 | 136 | 186 | 11774 | 40202 |
| 36 | 576 | 15156 | 36904 | 141 | 717 | 18413 | 98889 |
| 41 | 125 | 10883 | 35672 | 141 | 767 | 18337 | 47308 |
| 41 | 898 | 20306 | 34699 | 141 | 810 | 20274 | 47315 |
| 51 | 1123 | 24099 | 58044 |  |  |  |  |
| 52 | 951 | 20352 | 64171 |  |  |  |  |

**Supplementary Table 2.** Identification data (Center ID, Subject ID, SID, UID) of AD subjects involved in this study.

| **AD DATASET** | | | | | | | |
| --- | --- | --- | --- | --- | --- | --- | --- |
| **CENTER ID** | **SUBJECT ID** | **SID** | **UID** | **CENTER ID** | **SUBJECT ID** | **SID** | **UID** |
| 2 | 816 | 18402 | 40732 | 67 | 812 | 19629 | 38727 |
| 2 | 938 | 19852 | 40981 | 67 | 828 | 18532 | 65717 |
| 2 | 955 | 20004 | 40755 | 67 | 1185 | 24635 | 63105 |
| 3 | 1059 | 22300 | 52817 | 67 | 1253 | 27558 | 55034 |
| 3 | 1257 | 27340 | 52791 | 73 | 565 | 15762 | 39920 |
| 5 | 814 | 18390 | 74592 | 82 | 1377 | 28495 | 63171 |
| 7 | 1248 | 25568 | 59951 | 94 | 1102 | 22905 | 63187 |
| 7 | 1304 | 26475 | 59911 | 94 | 1164 | 23871 | 67224 |
| 11 | 3 | 9127 | 32238 | 94 | 1397 | 31011 | 95663 |
| 11 | 10 | 8800 | 32275 | 94 | 1402 | 32102 | 66079 |
| 13 | 592 | 18419 | 79145 | 98 | 884 | 24183 | 56027 |
| 13 | 699 | 18366 | 62651 | 99 | 470 | 14222 | 34571 |
| 13 | 1161 | 24399 | 51486 | 99 | 492 | 14944 | 38771 |
| 14 | 356 | 12857 | 39630 | 99 | 1144 | 24218 | 102041 |
| 14 | 1095 | 23323 | 45741 | 109 | 777 | 18676 | 82577 |
| 22 | 129 | 11485 | 59485 | 114 | 374 | 13031 | 39818 |
| 23 | 78 | 10619 | 52000 | 114 | 979 | 21933 | 39860 |
| 23 | 84 | 10764 | 31207 | 126 | 606 | 17191 | 38910 |
| 23 | 139 | 11079 | 31304 | 126 | 1221 | 25457 | 48977 |
| 27 | 1081 | 25357 | 47169 | 127 | 844 | 19874 | 34472 |
| 29 | 836 | 18151 | 65014 | 128 | 1409 | 33787 | 69401 |
| 29 | 1184 | 25463 | 67211 | 128 | 1430 | 39199 | 79858 |
| 33 | 889 | 19296 | 51630 | 130 | 1290 | 26038 | 63767 |
| 35 | 341 | 12952 | 45217 | 130 | 1337 | 27584 | 63776 |
| 36 | 577 | 14974 | 36915 | 131 | 457 | 13976 | 92407 |
| 36 | 760 | 18264 | 38653 | 131 | 497 | 15315 | 48039 |
| 57 | 474 | 13990 | 34721 | 131 | 691 | 17266 | 48048 |
| 57 | 1371 | 28667 | 62999 | 133 | 1170 | 24674 | 89958 |
| 57 | 1373 | 28698 | 63009 | 136 | 299 | 13839 | 40313 |
| 57 | 1379 | 28761 | 63015 | 136 | 300 | 14136 | 40329 |
| 62 | 535 | 14699 | 50427 | 136 | 426 | 14581 | 40357 |
| 62 | 690 | 16924 | 50469 | 141 | 1024 | 22699 | 47749 |
| 62 | 730 | 17062 | 50488 | 141 | 1137 | 24301 | 48582 |
| 67 | 29 | 9904 | 38718 | 141 | 1152 | 24487 | 48591 |
| 67 | 76 | 10468 | 35912 | 141 | 853 | 18348 | 112293 |
| 2 | 1018 | 23128 | 40818 | 33 | 1281 | 26136 | 54781 |
| 5 | 221 | 11958 | 72129 | 33 | 1283 | 26144 | 54786 |
| 5 | 929 | 19669 | 74610 | 33 | 1285 | 26128 | 51589 |
| 5 | 1341 | 27673 | 60418 | 33 | 1308 | 26600 | 54753 |
| 6 | 547 | 16033 | 67316 | 36 | 759 | 18094 | 36970 |
| 6 | 653 | 16073 | 67325 | 36 | 1001 | 22691 | 38662 |
| 7 | 316 | 12583 | 36574 | 41 | 1368 | 27512 | 65249 |
| 7 | 1339 | 27414 | 56320 | 41 | 1391 | 29116 | 62934 |
| 11 | 53 | 10064 | 35487 | 41 | 1435 | 39186 | 79637 |
| 11 | 183 | 12000 | 32004 | 51 | 1296 | 26431 | 58024 |
| 13 | 996 | 22240 | 51184 | 53 | 1044 | 21256 | 64204 |
| 13 | 1205 | 25024 | 51543 | 62 | 793 | 18189 | 50525 |
| 14 | 328 | 12402 | 39621 | 67 | 110 | 11177 | 35934 |
| 16 | 991 | 21737 | 40795 | 82 | 1079 | 22650 | 49491 |
| 16 | 1263 | 27303 | 64623 | 94 | 1027 | 21207 | 49528 |
| 20 | 213 | 12386 | 60601 | 94 | 1090 | 23375 | 63177 |
| 22 | 7 | 9024 | 59367 | 98 | 149 | 11021 | 89430 |
| 22 | 219 | 12375 | 59535 | 99 | 372 | 13672 | 34550 |
| 22 | 543 | 14849 | 59544 | 109 | 1157 | 24711 | 66159 |
| 23 | 83 | 10568 | 31144 | 109 | 1192 | 25056 | 63504 |
| 23 | 93 | 10736 | 31254 | 114 | 228 | 11697 | 49736 |
| 23 | 916 | 19228 | 31534 | 126 | 784 | 19752 | 39013 |
| 23 | 1262 | 26314 | 62434 | 126 | 891 | 19386 | 39055 |
| 23 | 1289 | 26374 | 89939 | 127 | 431 | 14595 | 34444 |
| 24 | 1171 | 24659 | 63407 | 127 | 754 | 18515 | 80761 |
| 24 | 1307 | 27061 | 63416 | 127 | 1382 | 28261 | 66311 |
| 27 | 404 | 13866 | 34205 | 130 | 956 | 20667 | 39187 |
| 27 | 850 | 18554 | 48997 | 130 | 1201 | 25082 | 63758 |
| 27 | 1254 | 25764 | 47229 | 133 | 1055 | 22386 | 40029 |
| 27 | 1385 | 28133 | 47575 | 136 | 194 | 13178 | 40240 |
| 29 | 999 | 23248 | 64899 | 141 | 696 | 18373 | 82739 |
| 29 | 1056 | 22977 | 60742 | 141 | 790 | 18766 | 91254 |
| 33 | 724 | 17337 | 42401 | 141 | 852 | 19395 | 47745 |
| 33 | 733 | 16932 | 42426 |  |  |  |  |

**Supplementary Table 3.** Identification data (Center ID, Subject ID, SID, UID) of MCIc subjects involved in this study.

| **MCIc DATASET** | | | | | | | |
| --- | --- | --- | --- | --- | --- | --- | --- |
| **CENTER ID** | **SUBJECT ID** | **SID** | **UID** | **CENTER ID** | **SUBJECT ID** | **SID** | **UID** |
| 2 | 954 | 19979 | 40745 | 33 | 906 | 19314 | 42469 |
| 2 | 1070 | 23120 | 40832 | 33 | 922 | 19341 | 42494 |
| 5 | 222 | 11754 | 54689 | 35 | 204 | 11661 | 39543 |
| 7 | 41 | 9994 | 35735 | 35 | 997 | 23184 | 62909 |
| 7 | 128 | 10936 | 36641 | 51 | 1331 | 29664 | 64153 |
| 7 | 344 | 12631 | 36580 | 52 | 952 | 20364 | 89953 |
| 11 | 856 | 19031 | 89409 | 52 | 1054 | 22955 | 62235 |
| 13 | 240 | 12308 | 51152 | 53 | 507 | 14483 | 80200 |
| 13 | 860 | 19237 | 51534 | 62 | 1299 | 26794 | 50585 |
| 22 | 750 | 17695 | 59553 | 67 | 243 | 12030 | 34820 |
| 22 | 1394 | 34317 | 68083 | 67 | 336 | 14023 | 34858 |
| 23 | 42 | 8852 | 31085 | 94 | 1015 | 21457 | 40764 |
| 23 | 388 | 13076 | 31438 | 94 | 1398 | 31771 | 63228 |
| 23 | 604 | 15182 | 31456 | 127 | 394 | 14603 | 34399 |
| 23 | 855 | 18561 | 31510 | 133 | 638 | 16608 | 67532 |
| 23 | 887 | 19087 | 31527 | 136 | 195 | 12523 | 40453 |
| 23 | 1247 | 25741 | 48858 | 141 | 982 | 22644 | 47704 |
| 27 | 461 | 15192 | 34232 | 941 | 1311 | 27408 | 97328 |
| 33 | 723 | 16845 | 42385 | 941 | 1363 | 28008 | 63898 |
| 33 | 725 | 17092 | 42410 | 941 | 1295 | 26290 | 63889 |
| 2 | 729 | 16874 | 40709 | 57 | 941 | 19985 | 34748 |
| 5 | 572 | 15709 | 32659 | 57 | 1217 | 25854 | 62985 |
| 6 | 1130 | 23457 | 55973 | 67 | 45 | 10185 | 35889 |
| 7 | 249 | 11911 | 36531 | 67 | 77 | 11136 | 68121 |
| 11 | 241 | 12088 | 32021 | 94 | 434 | 13570 | 39124 |
| 11 | 861 | 19476 | 35514 | 98 | 269 | 11964 | 65258 |
| 11 | 1282 | 26225 | 62637 | 99 | 54 | 10329 | 35826 |
| 13 | 325 | 13524 | 54666 | 99 | 111 | 10933 | 35850 |
| 14 | 658 | 17481 | 39702 | 126 | 1077 | 23496 | 48881 |
| 23 | 30 | 9441 | 31632 | 127 | 1427 | 37933 | 91127 |
| 23 | 625 | 15820 | 31496 | 128 | 947 | 19859 | 69074 |
| 27 | 179 | 11781 | 34137 | 130 | 423 | 15030 | 39134 |
| 27 | 256 | 12357 | 34151 | 133 | 727 | 18620 | 66358 |
| 27 | 1213 | 25492 | 47224 | 133 | 913 | 24646 | 63820 |
| 27 | 1387 | 28123 | 67202 | 136 | 695 | 19019 | 70925 |
| 33 | 567 | 14572 | 42371 | 141 | 915 | 20504 | 48573 |
| 41 | 549 | 15488 | 39507 | 141 | 1244 | 26845 | 92647 |
| 41 | 1412 | 34022 | 72220 |  |  |  |  |
| 41 | 1423 | 36902 | 72233 |  |  |  |  |

**Supplementary Table 4.** Identification data (Center ID, Subject ID, SID, UID) of MCInc subjects involved in this study.

| **MCInc DATASET** | | | | | | | |
| --- | --- | --- | --- | --- | --- | --- | --- |
| **CENTER ID** | **SUBJECT ID** | **SID** | **UID** | **CENTER ID** | **SUBJECT ID** | **SID** | **UID** |
| 3 | 1122 | 23542 | 52800 | 41 | 314 | 12492 | 34681 |
| 5 | 324 | 12599 | 32893 | 41 | 679 | 17077 | 40047 |
| 7 | 414 | 14826 | 36600 | 41 | 1010 | 23880 | 65231 |
| 11 | 326 | 12342 | 89391 | 41 | 1260 | 25806 | 65240 |
| 11 | 362 | 12678 | 89405 | 51 | 1072 | 22884 | 58012 |
| 11 | 1080 | 23159 | 35592 | 52 | 671 | 16062 | 64162 |
| 16 | 702 | 17341 | 40782 | 52 | 989 | 22476 | 64180 |
| 16 | 1028 | 22058 | 40800 | 52 | 1168 | 23688 | 65668 |
| 16 | 1138 | 24779 | 86046 | 53 | 621 | 15442 | 64190 |
| 22 | 1097 | 23337 | 59611 | 53 | 919 | 20422 | 65690 |
| 22 | 1351 | 28484 | 59616 | 57 | 464 | 14736 | 34708 |
| 23 | 126 | 11525 | 31272 | 57 | 1007 | 21339 | 47766 |
| 23 | 217 | 11731 | 31347 | 73 | 909 | 19716 | 75486 |
| 23 | 1046 | 22199 | 46397 | 94 | 1330 | 27038 | 63207 |
| 27 | 408 | 14231 | 37549 | 98 | 160 | 11224 | 65740 |
| 27 | 644 | 15630 | 34241 | 99 | 60 | 10478 | 35834 |
| 27 | 835 | 18760 | 35667 | 99 | 291 | 12065 | 34525 |
| 29 | 878 | 18986 | 64877 | 109 | 950 | 21165 | 97201 |
| 29 | 1038 | 22851 | 60733 | 114 | 378 | 12760 | 95689 |
| 29 | 1073 | 22828 | 65023 | 126 | 865 | 22295 | 39034 |
| 29 | 1215 | 25348 | 60747 | 126 | 1187 | 25143 | 48960 |
| 29 | 1218 | 25478 | 67390 | 127 | 112 | 11194 | 98858 |
| 33 | 511 | 15101 | 42241 | 127 | 1140 | 24278 | 63638 |
| 33 | 513 | 14673 | 42259 | 130 | 285 | 12462 | 39119 |
| 33 | 514 | 14663 | 42277 | 130 | 289 | 12111 | 39114 |
| 33 | 1279 | 26694 | 54758 | 130 | 449 | 22250 | 80931 |
| 33 | 1284 | 26686 | 54800 | 130 | 783 | 18023 | 39154 |
| 35 | 33 | 10396 | 45167 | 131 | 384 | 12790 | 48003 |
| 35 | 292 | 12408 | 39570 | 133 | 771 | 18575 | 92287 |
| 36 | 656 | 16286 | 36925 | 133 | 912 | 19884 | 40001 |
| 36 | 673 | 17157 | 36950 | 136 | 1227 | 26399 | 63839 |
| 36 | 748 | 17705 | 36960 | 141 | 697 | 18466 | 91236 |
| 36 | 869 | 21421 | 36994 | 141 | 851 | 19364 | 47871 |
| 36 | 976 | 23852 | 65092 | 141 | 1052 | 22923 | 47723 |
| 2 | 782 | 17835 | 40718 | 62 | 1182 | 25166 | 50567 |
| 2 | 1155 | 24144 | 40846 | 73 | 746 | 23286 | 63123 |
| 3 | 908 | 32516 | 62590 | 82 | 1119 | 23733 | 63148 |
| 3 | 1074 | 23536 | 53396 | 94 | 531 | 14554 | 49667 |
| 5 | 448 | 14032 | 32877 | 94 | 921 | 19582 | 49511 |
| 5 | 546 | 15566 | 32683 | 94 | 1293 | 27865 | 64375 |
| 5 | 1224 | 25412 | 60407 | 94 | 1314 | 27488 | 63197 |
| 7 | 101 | 10679 | 36727 | 98 | 667 | 15980 | 89496 |
| 7 | 293 | 12193 | 36550 | 99 | 51 | 10325 | 35820 |
| 7 | 698 | 16403 | 36614 | 99 | 1034 | 21759 | 47954 |
| 13 | 1186 | 25689 | 62657 | 109 | 1114 | 24702 | 63490 |
| 14 | 169 | 11565 | 40859 | 109 | 1183 | 24718 | 66168 |
| 14 | 557 | 15094 | 39675 | 114 | 410 | 13289 | 39825 |
| 14 | 563 | 16079 | 39693 | 114 | 458 | 14111 | 39846 |
| 16 | 769 | 17720 | 48868 | 114 | 1103 | 23239 | 49758 |
| 22 | 4 | 9234 | 64632 | 114 | 1106 | 22859 | 49911 |
| 22 | 544 | 14679 | 64673 | 114 | 1118 | 23803 | 49769 |
| 22 | 961 | 20711 | 59602 | 126 | 708 | 16897 | 38945 |
| 23 | 331 | 12428 | 31374 | 126 | 709 | 17326 | 38965 |
| 23 | 376 | 12652 | 31385 | 127 | 393 | 13200 | 34394 |
| 27 | 116 | 11442 | 34326 | 127 | 925 | 21560 | 67267 |
| 27 | 307 | 13072 | 34160 | 127 | 1032 | 22259 | 63633 |
| 27 | 485 | 14208 | 37558 | 128 | 1043 | 22562 | 69092 |
| 27 | 1045 | 22174 | 47211 | 130 | 102 | 10746 | 39461 |
| 33 | 1116 | 22799 | 42845 | 130 | 505 | 17292 | 39144 |
| 33 | 1309 | 26195 | 51606 | 133 | 629 | 15915 | 40955 |
| 36 | 945 | 20971 | 37000 | 133 | 792 | 18306 | 66374 |
| 36 | 1135 | 24501 | 65110 | 133 | 1031 | 21552 | 40020 |
| 36 | 1240 | 26423 | 65119 | 136 | 107 | 11707 | 40446 |
| 41 | 282 | 13496 | 39487 | 136 | 429 | 15534 | 40388 |
| 41 | 598 | 15604 | 40038 | 136 | 579 | 15952 | 40411 |
| 51 | 1131 | 24089 | 62944 | 136 | 874 | 22234 | 40420 |
| 53 | 389 | 13550 | 65677 |  |  |  |  |
| 57 | 839 | 19188 | 38671 |  |  |  |  |
